# Molecular Identification and Phylogenetic Analysis of Nuclear rDNA Sequences of *Clonorchis sinensis* Isolates From Human Fecal Samples in Heilongjiang Province, China

**DOI:** 10.3389/fmicb.2019.00026

**Published:** 2019-01-28

**Authors:** Xiaoli Zhang, Beibei Sun, Qiaoran Tang, Rui Chen, Su Han

**Affiliations:** ^1^Department of Parasitology, Harbin Medical University, Harbin, China; ^2^Department of Orthopaedics, The Fourth Affiliated Hospital of Harbin Medical University, Harbin, China

**Keywords:** *Clonorchis sinensis*, molecular identification, phylogenetic analysis, ITS1, ITS2, Heilongjiang Province

## Abstract

Studying the genetic diversity of parasite is important for understanding their biogeography and molecular epidemiology, as well as for establishing disease prevention and control strategies. *Clonorchis sinensis* is an important foodborne parasite worldwide. However, despite its epidemiological significance, the genetic diversity of *C. sinensis* has not been well studied from human in northeastern China. In this study, a total of 342 fecal specimens were collected from residents living in five villages in Heilongjiang Province and analyzed for the presence of *C. sinensis* by PCR amplification and sequencing of the internal transcribed spacer 1 (ITS1) and ITS2 regions of nuclear ribosomal DNA. 21.64% (74/342) of fecal samples were found to be positive for *C. sinensis* by PCR. The sequences of the ITS1 region in 34 of the 74 samples (45.95%) matched that of MK179278, Genetic polymorphisms were observed at six nucleotide sites. The ITS2 gene sequence of 37 of the 74 samples (50%) matched that of MK179281. In conclusion, a low degree of genetic diversity between *C. sinensis* isolates from China and different geographical regions was found at ITS loci. Despite this conservation, sequencing of the rDNA region has provided important data that will be useful for future studies addressing the molecular evolution, biology, medical implications and ecology of *C. sinensis.*

## Introduction

*Clonorchis sinensis* (*C. sinensis*) is an important foodborne zoonotic pathogen. Hosts become infected with *C. sinensis* by ingesting raw or undercooked freshwater fish containing metacercariae. Adults then parasitize the peripheral intrahepatic bile ducts. Typically, *C. sinensis* infections cause no obvious clinical symptoms or only mild symptoms (Zhang et al., 2008). However, high intensity or long-term *C. sinensis* infections can potentially lead to liver damage such as cholelithiases, cholecystitis and hepatic fibrosis (Choi et al., 2004). Furthermore, *C. sinensis* is considered a group I carcinogen-metazoan parasite that can potentially induce cholangiocarcinoma (Bouvard et al., 2009).

*Clonorchis sinensis* is endemic in Asia, particularly in China, Japan, Korea and Vietnam (Tang et al., 2016). In China, approximately 15 million people are estimated to be infected, mainly in southeast and northeast areas such as Guangdong, Guangxi and Heilongjiang Provinces (Lun et al., 2005). National sampling surveys showed that the prevalence of clonorchiasis in China increased by 75% from 1990 to 2003 and that Heilongjiang Province was an endemic focus (Rim, 2005; Tang et al., 2016). A study conducted in Heilongjiang Province between 2009 and 2012 found that the mean prevalence of *C. sinensis* was 25.93% (Han et al., 2013). However, despite its epidemiological significance, the genetic diversity of *C. sinensis* has not been sufficiently studied. Knowledge of genetic variation of *C. sinensis* is important for understanding its epidemiology and for disease control.

Molecular biology methods not only contribute to the understanding of parasite epidemiology but also allow for exploration of parasite characteristics, including host specificity, transmission patterns and genetic diversity (Choi et al., 2010; Huang et al., 2012). Researchers have investigated the genetic variation in *C. sinensis* isolates from Russia, Vietnam, China and Korea using molecular biology methods (Chelomina et al., 2014). Although *C. sinensis* is represented by a single species with a low divergence, karyotypic variation between *C. sinensis* isolates from China, Korea and eastern Russia suggests that this taxon might contain sibling species (Wang et al., 2017). To date, there have been no comprehensive studies exploring population genetic variation among large numbers of *C. sinensis* isolates from disparate geographic locations using complete or partial mitochondrial and/or nuclear genomic data sets.

Nuclear ribosomal DNA (rDNA) is widely used for molecular investigations and genetic analyses (Park, 2007). Ribosomal genes, including 18S, 5.8S and 28S rRNA, are typically organized into tandem repeats separated by two internal transcribed spacers (ITS1 and ITS2) (Prasad et al., 2008; Tatonova et al., 2017). Due to their high interspecific and low intraspecific variability, the ITS1 and ITS2 genes have been used extensively for ecological genetic studies and phylogenetic and evolutionary analyses at various taxonomic levels for different organisms (Dai et al., 2012; Buathong et al., 2017). Several studies have also been conducted on the genetic variability of trematode species such as *Schistosoma japonicum*, *Fasciola hepatica*, and *Opisthorchis felineus* (Katokhin et al., 2008; Zhao et al., 2010; Dar et al., 2012). However, limited information is available on the genetic diversity and molecular epidemiological surveys based on the ITS gene of *C. sinensis* from human in Heilongjiang Province.

In the present study, *C. sinensis* isolates from human feces in Heilongjiang Province were identified and genotyped by PCR amplification of ITS genes. The ITS1 and ITS2 gene sequences obtained here were compared with sequences previously published in GenBank. The sequence analysis were assessed by comparing the ITS1 and ITS2 gene sequences obtained here with those previously published in GenBank. These genetic data will be crucial for understanding the prevalence and genetic structure of *C. sinensis* and for developing disease treatment and control strategies.

## Materials and Methods

### Ethics Statement

This research study was approved by the Medical Ethics Review Committee of Harbin Medical University. The objectives, procedures and potential risks were explained to all participants. Written informed consent was obtained directly from all adult participants. If the participants were children, written informed consent were obtained from the next of kin, carers, or guardians on the behalf of the minors/children participants.

### Specimen Collection

**FIGURE 1:**
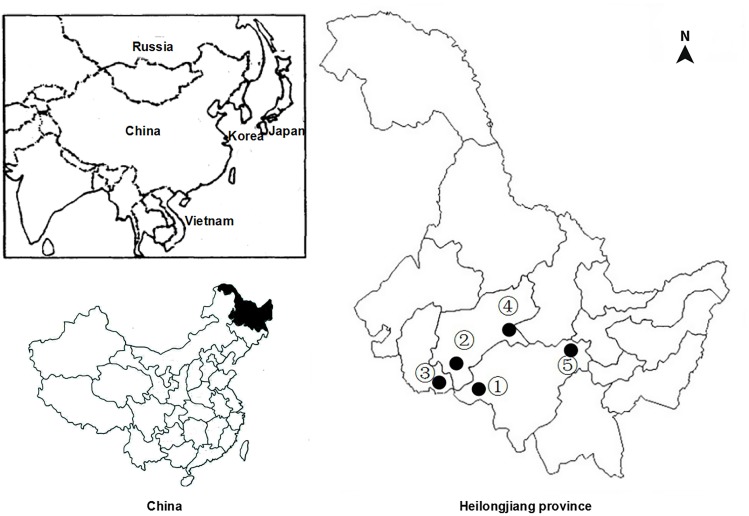
Sampling locations for *Clonorchis sinensis* in heilongjiang province, China. Numbers correspond with those in Table 1.

Between June 2014 and March 2015, a total of 342 fecal samples were collected from residents living five villages along Songhua river in Heilongjiang Province. Information about five villages is given in Figure 1. Approximately, 15–20 g of fecal specimens were randomly collected from each of participants. All fecal specimens were transported to the laboratory in a cooler with ice packs within 24 h and stored at 4°C until they were extracted.

### DNA Extraction

The fecal specimens were sieved and centrifuged at 1500 g for 10 minutes at room temperature, and then washed with distilled water for three times. Genomic DNA was extracted from 200 mg fecal specimen by a QIAamp DNA Stool Mini Kit (QIAgen, Hilden, Germany). The procedures and utilized reagents were utilized according to the manufacturer’s protocol. The eluted DNA was finally stored at -20°C in freezers until PCR analysis.

### PCR Amplification of the ITS1 and ITS2 Genes

Each of the DNA specimens was detected for the presence of *C. sinensis* by amplifying 572 bp of the ITS1 gene and 249 bp of the ITS2 gene, respectively. The primes for ITS1 are CsITS1F (5′-CGATTCTAGTTCCGTCATCT-3′) and CsITS1R (5′-CCGCTCAGAGTACTCAT-3′) (Liu et al., 2007). The primers of the ITS2 gene are CsITS2F (5′-TATAAACTATCACGACGCCC-3′) and CsITS2R (5′-TACTGAAGCCTCAACCAAAG-3′) (Yang et al., 2014).

Amplification of the ITS1 gene was used the following cycling conditions: a 4 min initial denaturation step at 95°C; 35 cycles of 30 s at 94°C, 1 min at 62°C, 1 min at 72°C; and a 10 min extension at 72°C. The cycling parameters of amplification of the ITS2 gene were as follows: a 3 min initial denaturation step at 95°C; 35 cycles of 30 s at 95°C, 30 s at 55°C, 60 s at 72°C; and a 3 min extension at 72°C. Negative and positive controls with both primers were included, respectively. TaKaRa Taq DNA polymerase (TaKaRa Bio Inc., Tokyo, Japan) was used for all the PCR amplifications. Then the PCR products were separated in 1.5% agarose gel electrophoresis and visualized under UV light after staining with ethidium bromide.

### Nucleotide Sequencing

All the PCR products of expected size were directly sequenced on an ABI PRISM 3730 XL DNA Analyzer by Sinogeno-max Biotechnology Co., Ltd. (Beijing, China), using the BigDye Terminator v3.1 Cycle Sequencing Kit (Applied Biosystems, Foster City, CA, United States).

### DNA Sequence Analysis

The positive results in electrophoresis were selected for sequencing. All the nucleotide sequences were aligned with each other. According to the identity percentage and query coverage parameter, the reference sequences downloaded from GenBank database using the Basic Local Alignment Search Tool (BLAST)^1^ and Clustal X 1.83^2^ to determine isolates of *C. sinensis*.

### Nucleotide Sequence Accession Numbers

If the isolates obtained in this study with the cutoff values of above 95% in sequence similarity were identical to those published in GenBank, they were identified to be known isolates and given the first published name. If not, they were considered to be novel isolates. Representative nucleotide sequences obtained in the present study were deposited in the GenBank database under accession numbers MK179278 to MK179280 (ITS1), MK179281 to MK179283 (ITS2).

### Date Analysis

To better present the diversity of all the isolates of *C. sinensis* obtained in this study and to assess the genetic relationship of the novel ones here to the known ones, intraspecific phylogenies were reconstructed with the neighbor-joining (NJ) and maximum likelihood (ML) methods in MEGA7.0 program. NJ and ML trees were constructed using 1000 and 100 bootstrap replicates, respectively. In addition, Bayesian analysis was also used and proceed as follow: the posterior probabilities are determined by Markov chain Monte Carlo sampling (MCMC) in MrBayes v3.2 (Ronquist et al., 2012) based on the models from MrModeltest (Posada and Crandall, 1998; Posada, 2008). There were 275000 generation for ITS1 and 170000 generation for ITS2 run in MrBayes program. An average standard deviation of <0.01 for split frequencies is used to suggest a convergence between parallel runs. Twenty-five percent of the total trees are discarded as burn-in. Additional, the network was also generated with SplitsTree (SplitsTree 4.0 program) using the Neighbor Network method to display the samples (Morrison, 2005; Huson and Bryant, 2006).

The ITS1 sequences of *Metorchis bilis* (KY356536), *M.xanthosomus* (KY356540), *M.orientalis* (KX832894), *M.orientalis* (KX857496), *Opisthorchis felineus* (EU038139), *O. felineus* (KR995729), and *O. felineus* (KT020830) were used as the outgroup. The ITS2 sequences of *M.bilis* (KT740982), *M.orientalis* (KX857496), *M.xanthosomus* (KT740977), *O. noverca* (KJ767634), *O. lobatus* (HQ328545) and *O. parageminus* (KX258657) were chosen as the outgroup to root the trees. The appropriate nucleotide substitution model for each gene is determined using MrModeltest MrModeltest v2.3 (Nylander, 2008). The Kimura-2-parameter “K2” model is the best model for both ITS1 and ITS2 sequence data.

SPSS (version 10.0 software for windows; Chicago, IL, United States) was used for analyzing the date. The chi-square test was used to evaluate the assessment between qualitative variable to check for statistical differences. *P* < 0.05 was regarded as statistically significant.

## Results

### Prevalence of *C. sinensis*

In the present study, a total of 342 human fecal specimens were examined for *C. sinensis* by PCR amplification of the ITS1 and ITS2 genes. *C. sinensis* was found in all five villages examined, with infection rates ranging from 12.31 to 33.33% (Table 1 and Figure 1). Specimens that were found to be positive for *C. sinensis* by PCR were confirmed by sequencing, and an overall infection rate of 21.64% was found (74/342).

**Table 1:** Regional distribution of *C. sinensis* infection in five localities from Heilongjiang Province based on ITS gene.

Locality		No. of	No. of	Percentage
number	Geographic origin	positive	examined	(%)
1	Harbin Northeast	28	97	28.87
2	Suihua West	10	51	19.61
3	Daqing	19	57	33.33*
4	Suihua Northeast	8	65	12.31
5	Harbin West	9	72	12.50
Total		74	342	21.64


*^∗^P < 0.05, significantly different from these five locality.*

### Genetic Characterization of the *C. sinensis* ITS1 and ITS2 Gene

The ITS1 gene sequence of 34 of the 74 samples (45.95%) matched that of MK179278, followed by MK179280 (25.68%, 19/74), MK179279 (24.32%, 18/74), and MF319641, HQ874526, JQ048598 (one each, 1.35%, 1/74) (Table 2). The genetic polymorphisms of one to two base variations were observed at six nucleotide sites (Table 3).

**Table 2:** ITS1 sequences of *C. sinensis* from Heilongjiang Province in this study.

Gene	GenBank accession number	*n*	Percentage (%)
ITS1	MK179278	34	45.95
	MK179280	19	25.68
	MK179279	18	24.32
	HQ874526	1	1.35
	JQ048598	1	1.35
	MF319641	1	1.35
	Total	74	


**Table 3:** Nucleotide variation at six polymorphic sites in ITS1 gene region of *C. sinensis* isolates from human in this study.

GenBank accession number (n)	Nucleotide at position (ITS1)					
	114	507	518	525	531	590
MF319655	Y	G	–	C	G	G
MK179278(34)	–	G	–	C	G	G
MK179280(19)	–	G	–	C	G	G
MK179279(18)	–	G	–	C	G	G
HQ874526(1)	–	G	–	A	T	C
JQ048598(1)	C	C	G	C	G	G
MF319641(1)	Y	C	T	C	G	G


The ITS2 gene sequence of 37 of the 74 samples (50%) matched that of MK179281, followed by MK179283 (25.68%, 19/74) and MK179282 (24.32%, 18/74), respectively. There were no genetic polymorphisms observed.

### Phylogenetic Relationships of *C. sinensis*

The phylogenetic analysis was carried out based on the ITS gene sequences of the isolates of *C. sinensis* and some isolates published previously, inferred by neighbor-joining (NJ), maximum likelihood (ML) and Bayesian (Bayes) analyses, with *M. bilis, M. xanthosomus, M. orientalis, O. lobatus, O. felineus, O. noverca, and O. parageminus* as outgroup.

**FIGURE 2:**
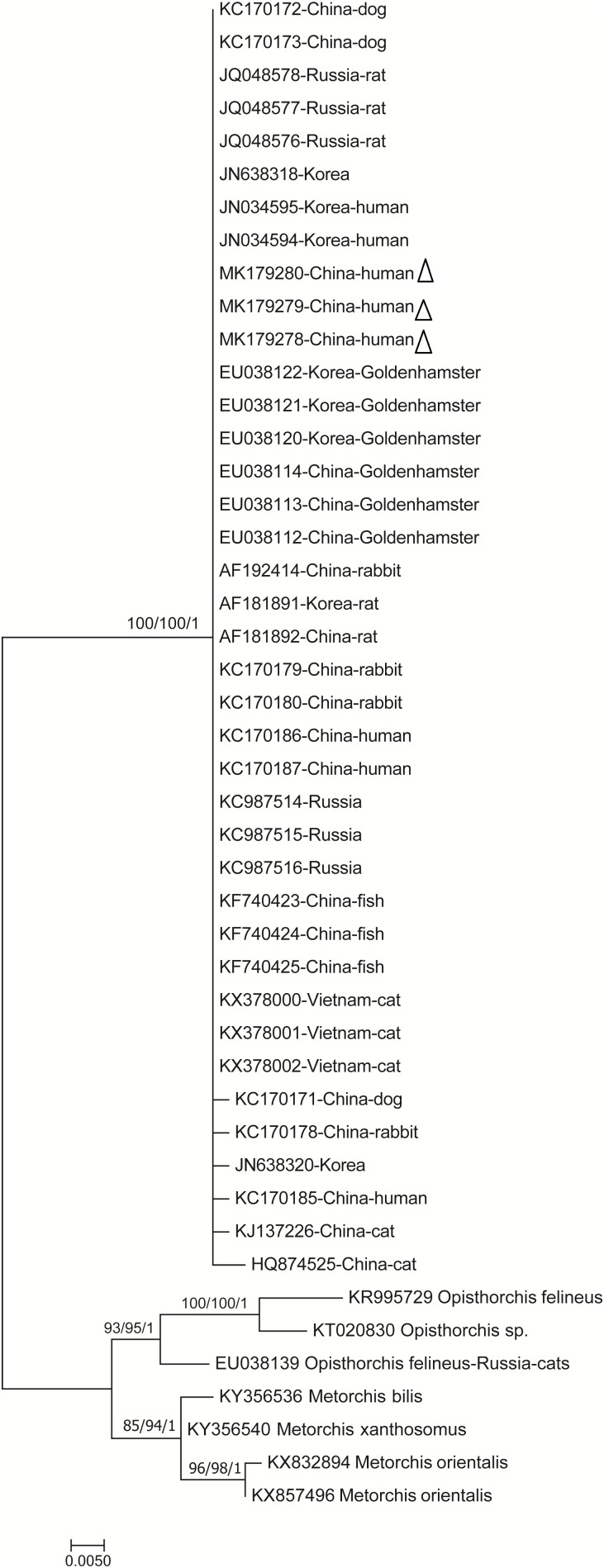
Phylogenetic relationships between the *C. sinensis* isolates based on the partial ITS1 rDNA sequences. Nodal support of >50% is shown for maximum likelihood/neighbor-joining/Bayesian Inference analyses (as indicated). *Opisthorchis felineus* and *Metorchis bilis* et al. were used for comparison. Each sequence is identified by its accession number and host origin. Genotypes with black triangle are genotypes identified in this study, respectively.

**FIGURE 3:**
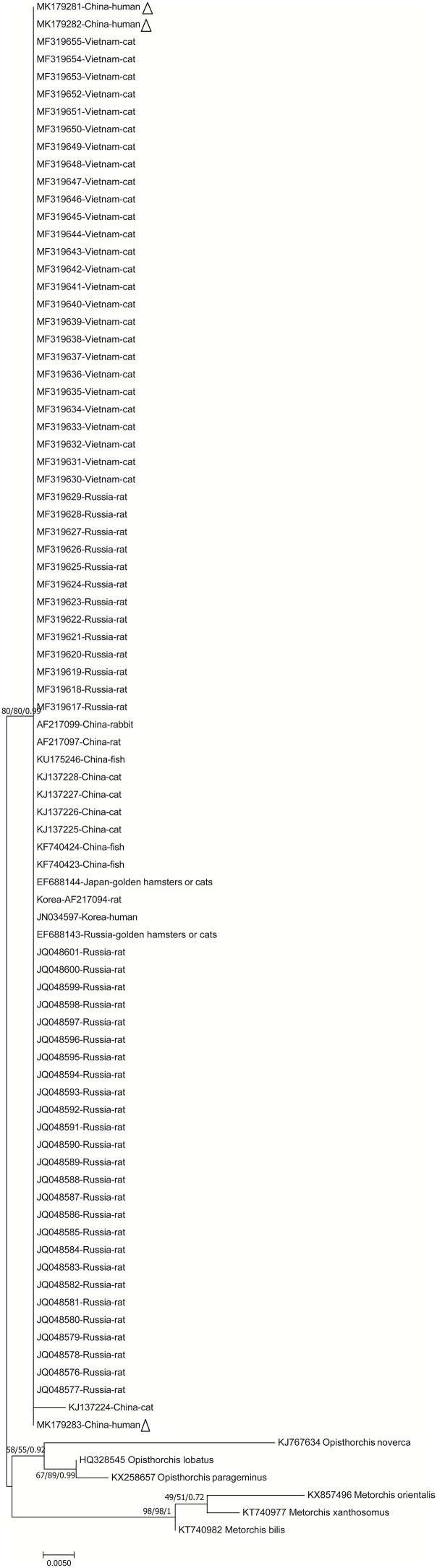
Phylogenetic relationships between the *C. sinensis* isolates based on the partial ITS2 rDNA sequences. Nodal support of >50% is shown for maximum likelihood/neighbor-joining/Bayesian Inference analyses (as indicated). *Opisthorchis noverca and Opisthorchis lobatus* et al. were used for comparison. Each sequence is identified by its accession number and host origin. Genotypes with black triangle are genotypes identified in this study, respectively.

The topologies of NJ, ML and Bayesian (Figure 2, 3) trees were very similar and only small differences in bootstrap support values were obtained among those. These trees demonstrated unresolved topology with low bootstrap support for most branches. None of those corresponded to the geographical localities based on ITS1 gene; nevertheless, only a few clusters were statistically supported according to the geographical distance based on ITS2 gene. Additional, the Neighbor Network was also used to display the samples (Supplementary Figures S1, S2). Instead of conventional single phylogenetic tree, Splits Tree makes a phylogenetic network with reticulations. It also showed that *C. sinensis* isolates clustered together with the exclusion of other liver fluke representatives.

## Discussion

Genetic data are crucial for understanding the biological history of parasitic diseases and developing treatment and control strategies (Gasser and Newton, 2000). In addition, genetic variation is common in parasite populations and is a valuable resource for studying the population biology, epidemiology, and genetic structure of parasites (Liu et al., 2012). Although intraspecific and interspecific variations between *C. sinensis* from different geographic regions have recently been studied using nuclear rDNA and mitochondrial DNA sequences (Park, 2007; Sun et al., 2013), there is a paucity of information on the genetic variation among *C. sinensis* populations from disparate geographic locations, where *C. sinensis* infection remains a significant health problem (Han et al., 2016).

In the present study, ITS genes were targeted to detect genetic variations in *C. sinensis* isolates from human fecal samples collected in five villages in Heilongjiang Province, China. We found that the *C. sinensis* infection rate was 21.64%. In contrast, the prevalence of *C. sinensis* infection in Korea was from 1.6 to 9.0% during 1993 – 2006 (Kim et al., 2010). Infection rates are related to many factors, including the sensitivity and specificity of the detection method, infection intensity, overall sample size, climate and experimental design (Tang et al., 2016). The high infection rate in the present study may have been due to the age group, sex, or other demographic factors of the individuals assessed (Han et al., 2013; Lai et al., 2017). For example, males prefer to eat more fish and have more frequent social activities and eating opportunities at restaurants compared with females (Lun et al., 2005). This results in a higher infection rate among males than females. Prevalence rates also increased with age, reaching a plateau among adolescents and young adults and decreasing in elderly people (Han et al., 2013). As an *C.sinensis* endemic area, Heilongjiang Province has numerous rivers, including the Songhua River, which is an important site for carp, grass carp, and catfish and for the freshwater fish industry (Han et al., 2013). The consumption of raw or undercooked freshwater fish and/or shrimp has been prevalent in this area for a long time, and it is difficult to change eating habits in the short time (Qian et al., 2016). Thus, further prevention and control strategies should be strengthened in these villages, especially the promotion of public health among local residents (Guoqing et al., 2001).

In the present study, 74 *C. sinensis* samples were distributed among six genotypes based on their ribosomal ITS1 region sequences. Additionally, six variations in the ITS1 gene region were observed in this study. Nucleotide diversity within ITS1 region was unequally distributed and mainly located at the ends of the sequence. Similarly, another study of genetic variation within *C. sinensis* in Korea and China also demonstrated a high degree of similarity in the ITS rDNA sequences (Lee and Huh, 2004). In trematodes, including *C. sinensis*, intraspecific variation in the ITS sequences is minimal (Liu et al., 2007; Xiao et al., 2013). This low genetic diversity may be due to high levels of gene flow between parasite populations, which counteracts local adaptation (Sun et al., 2013). However, the small number of polymorphisms in the ITS sequence (one locus) does not necessarily mean that there was no genetic differentiation. Analysis of the rDNA region has provided new data that will be useful for future studies addressing the molecular evolution, biology, and ecology of *C. sinensis* (Tatonova et al., 2017). Indeed, due to selective pressures from the human host, many parasites, such as *Giardia* species, have low levels of genetic variation. The features of the ITS region might reflect an adaptation strategy of *C. sinensis* to environmental conditions and different hosts. No variations in length or nucleotide composition were detected in the ITS2 region of three genotypes in this study. The lack of intraspecific variability in the ITS2 region was also confirmed by a genetic diversity study conducted in eastern Russian (Tatonova et al., 2012). Therefore, ITS2 might be a suitable and sensitive marker for species-level analysis (Tatonova et al., 2017).

Phylogenetic analysis was used to assess the genetic relationships of *C. sinensis*. The phylogenetic tree of intraspecific and interspecific relationships based on the ITS1 and ITS2 sequences showed low divergence among all tested isolates. Phylogenetic trees constructed using the NJ, ML and Bayesian analyze and Neighbor Network showed that all *C. sinensis* isolates clustered together with the exclusion of other liver fluke representatives. Nonetheless, the clustering within *C. sinensis* was not well supported in the analyses. Such information can be useful to improve our understanding of the molecular mechanisms of species adaptation and evolution and parasite infection strategies (Chelomina et al., 2014). ITS rDNA spacers usually diverge among species but are homogeneous within species due to concerted evolution (Bower et al., 2008). Nevertheless, we believe that these genetic data could have important epidemiological, evolutionary and medical implications.

Based on the analysis of ITS region form GenBank sequences, this was the first genetic analysis based on ITS1 and ITS2 sequences of *C. sinensi*s from human in different regions of Heilongjiang Province (Supporting Information Supplementary Tables S1–S4). The results could provide an important reference for future studies on *C. sinensis*, including species identification studies and assessments of molecular variations between disparate geographical locations. Most importantly, our research will be valuable for the further classification and identification of *C. sinensis* for the purposes of developing suitable disease prevention and control strategies.

There were some limitations to this study. Firstly, the number of fecal samples collected for this study was relatively small, and our findings were potentially related to the limited number of specimens. Although collection of fecal samples from humans can be difficult, more extensive investigations with a larger number of animal and human specimens are required in the future. Secondly, future studies analyzing other molecular markers are necessary to provide additional information on the population genetic structure of this parasite.

## Conclusion

In conclusion, this study provided molecular evidence of *C. sinensis* in northeast China. A low degree of genetic diversity between *C. sinensis* isolates from China and different geographical regions was found at ITS loci. The rDNA region data has provided important information that will be useful for future studies addressing the molecular evolution, biology, medical implications, and ecology of *C. sinensis.* In the future, analysis of additional samples and molecular markers is necessary to provide further information on the population genetic structure of *C. sinensis*, enhancing our ability to prevention and control strategies of *C. sinensis.*

## Author Contributions

SH and XZ conceived and designed the experiments. SH, BS, QT, and XZ performed the experiments. SH and XZ analyzed the data. SH, BS, QT, and RC contributed to reagents, materials, and analysis tools. SH, BS, and XZ wrote the paper. All authors edited the manuscript, read, and approved the final version of the manuscript.

## Conflict of Interest Statement

The authors declare that the research was conducted in the absence of any commercial or financial relationships that could be construed as a potential conflict of interest.

## Footnotes

1 http://blast.ncbi.nlm.nih.gov/Blast.cgi

2 http://www.clustal.org/

## Supplementary Material

FIGURE S1 The phylogenetic split network of the *Clonorchis sinensis* isolates based on the partial ITS1 rDNA sequences. Genotypes with black triangle are genotypes identified in this study, respectively.

FIGURE S2 The phylogenetic split network of the *Clonorchis sinensis* isolates based on the partial ITS2 rDNA sequences. Genotypes with black triangle are genotypes identified in this study, respectively.

Table S1 ITS1 sequences of *C. sinensis* worldwide.

Table S2 ITS1 sequences of *C. sinensis* by geographical location in China.

Table S3 ITS2 sequences of *C. sinensis* worldwide.

Table S4 ITS2 sequences of *C. sinensis* by geographical location in China.
