# Supplementary material for: Molecular Identification and Phylogenetic Analysis of Nuclear rDNA Sequences of Clonorchis sinensis Isolates From Human Fecal Samples in Heilongjiang Province, China
Source: Front Microbiol. 2019 Jan 28;10:26. doi: 10.3389/fmicb.2019.00026 (PMC6360181; doi:10.3389/fmicb.2019.00026)
Supplement: Table S1 — ITS1 sequences of C. sinensis worldwide. [file Table_1.DOCX]

Table S1. ITS1 sequences of *C. sinensis* worldwide

| Country | City | Host | Stage | GenBank accession number | n | Reference |
| --- | --- | --- | --- | --- | --- | --- |
| [Russia](D:/Program%20Files/Youdao/Dict/6.3.69.8341/resultui/frame/javascript:void(0);) | Kronshtadtka(KR) | rat | adult | JQ048576-JQ048588,  JQ048602-JQ048621 | 33 | Tatonova *et al.*, 2012 |
|  | Kondratenovka(KO) | rat | adult | JQ048589-JQ048601 | 13 | Tatonova *et al.*, 2012 |
|  | Magdikovoe(MA) | rat | adult | MF319617-MF319629 | 13 | Tatonova et al., 2017 |
|  | Khabarovsk Kray(KH) | / | / | KC987527-KC987539 | 13 | unpublished |
|  | PrimKr(PRI) | / | / | KC987519-KC987526 | 8 | unpublished |
|  | Primorsky Krai(PRK) | / | / | KC987514-KC987518 | 5 | unpublished |
| Vietnam | unknow(None) | cat | adult | KX378000-KX378009  KX377980- KX377999 | 30 | unpublished |
|  | Thai Binhan(TB) | cat | adult | MF319630-MF319642 | 13 | Tatonova *et al*., 2017 |
|  | Danang(DA) | cat | adult | MF319643-MF319655 | 13 | Tatonova *et al*., 2017 |
| Korea | Gyeongsangnam-do(GND) | Golden hamster | adult | EU038120-EU038122, EU038126-EU038133 | 11 | Kang *et al*., 2008 |
|  | Jeollanam-do(JND) | Golden hamster | adult | EU038123-EU038125 | 3 | Kang *et al*., 2008 |
|  | South Korea(SK) | / | / | JN638318, JN638320 | 2 | unpublished |
|  |  | rat | adult | AF181891 | 1 | Lee *et al*., 2004 |
|  | Gyeongsangbuk-do(GBD) | human | egg | JN034594, JN034595 | 2 | unpublished |
| Total |  |  |  |  | 160 |  |

/ unkown the host and stage

Table S2 ITS1 sequences of *C. sinensis* by geographical location in China

| Province | Host | Stage | GenBank accession number | n | Reference |
| --- | --- | --- | --- | --- | --- |
| Liaoning(LN) | Golden hamster | adult | EU038112-EU038119 | 8 | Kang *et al.* 2008 |
|  | rabbit | adult | AF192414 | 1 | Lee *et al.*, 2004 |
| Guangxi(GX) | cat | adult | HQ874525,HQ874529, HQ874533, | 8 | Sun *et al.* 2011 |
|  |  |  | HQ874536,HQ874542, HQ874543, |  |  |
|  |  |  | HQ874579, HQ874580 |  |  |
|  | dog | adult | KC170171-KC170177 | 7 | Xiao *et al.* 2013 |
|  | cat | adult | KJ137224-KJ137226 | 3 | unpublished |
|  | rat | adult | AF181892 | 1 | Lee *et al.*, 2004 |
| Guangdong(GD) | cat | adult | HQ874532,HQ874535, HQ874540, | 8 | Sun *et al.* 2011 |
|  |  |  | HQ874541,HQ874581, HQ874582, |  |  |
|  |  |  | HQ874587, HQ874602 |  |  |
|  | cat | adult | KC170164-KC170170 | 7 | Xiao *et al.* 2013 |
| Hubei(HB) | cat | adult | HQ874524, HQ874539, | 9 | Sun *et al.* 2011 |
|  |  |  | HQ874544-HQ874547, HQ874578, |  |  |
|  |  |  | HQ874592, HQ874593 |  |  |
| Anhui(AH) | cat | adult | HQ874523,HQ874537, HQ874538, | 8 | Sun *et al.* 2011 |
|  |  |  | HQ874584-HQ874586, HQ874588, |  |  |
|  |  |  | HQ874599 |  |  |
| Henan(HeN) | cat | adult | HQ874550, HQ874571-HQ874573, | 8 | Sun *et al.* 2011 |
|  |  |  | HQ874589,HQ874595, HQ874597, |  |  |
|  |  |  | HQ874601 |  |  |
| Hunan(HuN) | cat | adult | HQ874534, HQ874551-HQ874554, | 8 | Sun *et al.* 2011 |
|  |  |  | HQ874590,HQ874591, HQ874598 |  |  |
| Jiangsu(JS） | cat | adult | HQ874530,HQ874558, HQ874559, | 7 | Sun *et al.* 2011 |
|  |  |  | HQ874566,HQ874569,HQ874583, HQ874604 |  |  |
| Jiangxi(JX) | cat | adult | HQ874560, HQ874561, | 6 | Sun *et al.* 2011 |
|  |  |  | HQ874563-HQ874565, HQ874596 |  |  |
| Zhejiang(ZJ) | / | adult | HQ874531,HQ874562, HQ874567, | 6 | Sun *et al.* 2011 |
|  |  |  | HQ874568,HQ874594,HQ874600 |  |  |
| Jilin(JL) | dog | adult | HQ874527, HQ874528, | 6 | Sun *et al.* 2011 |
|  |  |  | HQ874555-HQ874557, HQ874570 |  |  |
|  | / | adult | HQ186260 | 1 | unpublished |
| Heilongjiang(HLJ) | / | adult | HQ874526,HQ874548, HQ874549, | 8 | Sun *et al.* 2011 |
|  |  |  | HQ874574-HQ874577, HQ874603 |  |  |
|  | / | adult | HQ186253-HQ186259 | 7 | unpublished |
|  | rabbit | adult | KC170178-KC170184 | 7 | Xiao *et al.* 2013 |
|  | human | adult | KC170185-KC170191 | 7 | Xiao *et al.* 2013 |
|  | fish | metacercaria | KF740423-KF740425 | 3 | Zhang *et al.*, 2014 |
| Total |  |  |  | 134 |  |

Table S3 ITS2 sequences of *C. sinensis* worldwide

| Country | City | Host | Stage | GenBank accession number | n | Reference |
| --- | --- | --- | --- | --- | --- | --- |
| Russia | Kronshtadtka(KR) | rat | adult | JQ048576-JQ048588 | 13 | Tatonova *et al.*, 2012 |
|  | Kondratenovka(KO) | rat | adult | JQ048589-JQ048601 | 13 | Tatonova *et al*., 2012 |
|  | Magdikovoe(MA) | rat | adult | MF319617-MF319629 | 13 | Tatonova *et al*., 2017 |
|  | Khabarovsk Kray(KH) | golden hamsters or cats | adult | EF688143 | 1 | Katokhin *et al*., 2008 |
| Korea | Gyeongsangbuk-do(GBD) | human | egg | JN034597 | 1 | unpublished |
|  | Kimhae(KI) | rat | adult | AF217094 | 1 | Lee *et al*., 2004 |
| Japan | Kurashiki(KU) | golden hamsters or cats | maritae | EF688144 | 1 | Katokhin *et al.*, 2008 |
| Vietnam | Thai Binhan(TB) | cat | adult | MF319630-MF319642 | 13 | Tatonova *et al*., 2017 |
|  | Danang(DA) | cat | adult | MF319643-MF319655 | 13 | Tatonova *et al*., 2017 |
| Total |  |  |  |  | 69 |  |

Table S4 ITS2 sequences of *C. sinensis* by geographical location in China

| Province | Host | Stage | GenBank accession number | n | Reference | |
| --- | --- | --- | --- | --- | --- | --- |
| Heilongjiang(HLJ) | fish | metacercaria | KF740423-KF740425 | 3 | Zhang *et al*., 2014 | |
| Guangxi(GX) | cat | adult | KJ137224-KJ137226 | 3 | unpublished | |
|  | cat | metacercaria | KJ137227-KJ137228 | 2 | unpublished | |
|  | fish | metacercaria | KU175246 | 1 | unpublished | |
|  | rat | adult | AF217097 | 1 | Lee *et al*., 2004 |  |
| Liaoning(LN) | rabbit | adult | AF217099 | 1 | Lee *et al*., 2004 | |
| Total |  |  |  | 11 |  | |
