# Supplementary material for: Molecular Identification and Phylogenetic Analysis of Nuclear rDNA Sequences of Clonorchis sinensis Isolates From Human Fecal Samples in Heilongjiang Province, China
Source: Front Microbiol. 2019 Jan 28;10:26. doi: 10.3389/fmicb.2019.00026 (PMC6360181; doi:10.3389/fmicb.2019.00026)
Supplement: FIGURE S1 — The phylogenetic split network of the Clonorchis sinensis isolates based on the partial ITS1 rDNA sequences. Genotypes with black triangle are genotypes identified in this study, respectively. [file Data_Sheet_1.PDF]

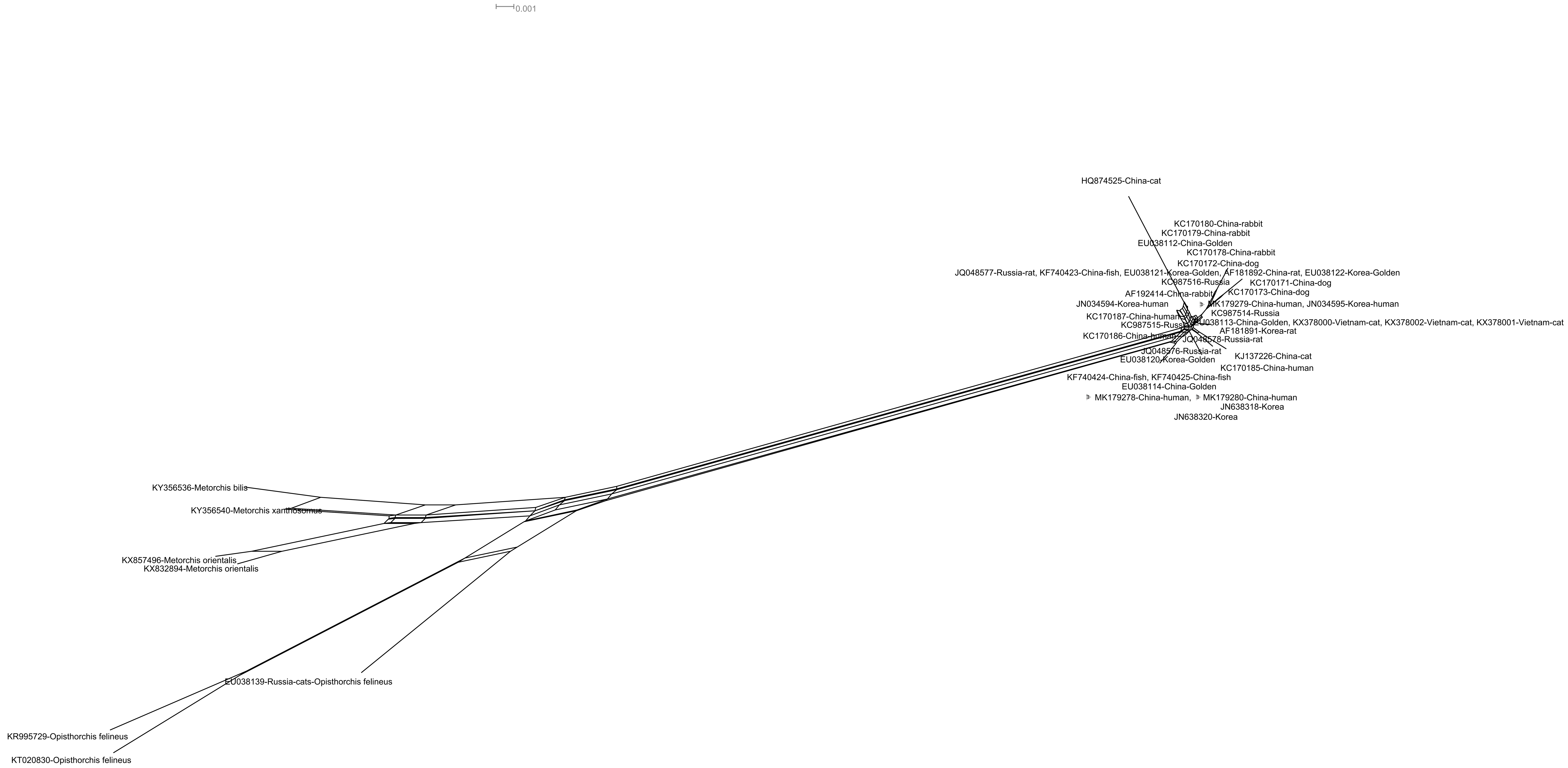
0.001
HQ874525-China-cat
KC170180-China-rabbit
KC170179-China-rabbit
EU038112-China-Golden
KC170178-China-rabbit
KC170172-China-dog
JQ048577-Russia-rat, KF740423-China-fish, EU038121-Korea-Golden, AF181892-China-rat, EU038122-Korea-Golden
KC987516-Russia
KC170171-China-dog
KC170173-China-dog
AF192414-China-rabbit
JN034594-Korea-human
MK179279-China-human, JN034595-Korea-human
KC987514-Russia
KC170187-China-human
KC987515-Russia
EU038113-China-Golden, KX378000-Vietnam-cat, KX378002-Vietnam-cat, KX378001-Vietnam-cat
AF181891-Korea-rat
KC170186-China-human
JQ048578-Russia-rat
JQ048576-Russia-rat
EU038120-Korea-Golden
KJ137226-China-cat
KC170185-China-human
KF740424-China-fish, KF740425-China-fish
EU038114-China-Golden
MK179278-China-human,
MK179280-China-human
JN638318-Korea
JN638320-Korea
KY356536-Metorchis bilis
KY356540-Metorchis xanthosomus
KX857496-Metorchis orientalis
KX832894-Metorchis orientalis
EU038139-Russia-cats-Opisthorchis felineus
KR995729-Opisthorchis felineus
KT020830-Opisthorchis felineus
