# Supplementary material for: Molecular Identification and Phylogenetic Analysis of Nuclear rDNA Sequences of Clonorchis sinensis Isolates From Human Fecal Samples in Heilongjiang Province, China
Source: Front Microbiol. 2019 Jan 28;10:26. doi: 10.3389/fmicb.2019.00026 (PMC6360181; doi:10.3389/fmicb.2019.00026)
Supplement: FIGURE S2 — The phylogenetic split network of the Clonorchis sinensis isolates based on the partial ITS2 rDNA sequences. Genotypes with black triangle are genotypes identified in this study, respectively. [file Data_Sheet_2.PDF]

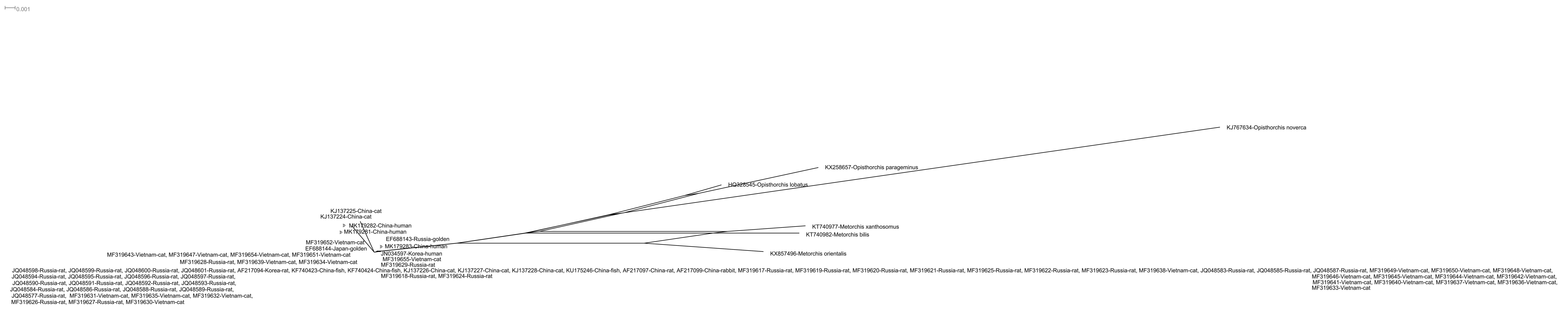
0.001
KJ767634-Opisthorchis noverca
KX258657-Opisthorchis parageminus
HQ328545-Opisthorchis lobatus
KT740977-Metorchis xanthosomus
KT740982-Metorchis bilis
KX857496-Metorchis orientalis
KJ137225-China-cat
KJ137224-China-cat
MK179282-China-human
MK179281-China-human
EF688143-Russia-golden
MF319652-Vietnam-cat
MK179283-China-human
EF688144-Japan-golden
MF319643-Vietnam-cat, MF319647-Vietnam-cat, MF319654-Vietnam-cat, MF319651-Vietnam-cat
JN034597-Korea-human
MF319655-Vietnam-cat
MF319628-Russia-rat, MF319639-Vietnam-cat, MF319634-Vietnam-cat
MF319629-Russia-rat
JQ048598-Russia-rat, JQ048599-Russia-rat, JQ048600-Russia-rat, JQ048601-Russia-rat, AF217094-Korea-rat, KF740423-China-fish, KF740424-China-fish, KJ137226-China-cat, KJ137227-China-cat, KJ137228-China-cat, KU175246-China-fish, AF217097-China-rat, AF217099-China-rabbit, MF319617-Russia-rat, MF319619-Russia-rat, MF319620-Russia-rat, MF319621-Russia-rat, MF319625-Russia-rat, MF319622-Russia-rat, MF319623-Russia-rat, MF319638-Vietnam-cat, JQ048583-Russia-rat, JQ048585-Russia-rat, JQ048587-Russia-rat, MF319649-Vietnam-cat, MF319650-Vietnam-cat, MF319648-Vietnam-cat,
MF319618-Russia-rat, MF319624-Russia-rat
JQ048594-Russia-rat, JQ048595-Russia-rat, JQ048596-Russia-rat, JQ048597-Russia-rat,
JQ048590-Russia-rat, JQ048591-Russia-rat, JQ048592-Russia-rat, JQ048593-Russia-rat,
JQ048584-Russia-rat, JQ048586-Russia-rat, JQ048588-Russia-rat, JQ048589-Russia-rat,
JQ048577-Russia-rat, MF319631-Vietnam-cat, MF319635-Vietnam-cat, MF319632-Vietnam-cat,
MF319626-Russia-rat, MF319627-Russia-rat, MF319630-Vietnam-cat
MF319646-Vietnam-cat, MF319645-Vietnam-cat, MF319644-Vietnam-cat, MF319642-Vietnam-cat,
MF319641-Vietnam-cat, MF319640-Vietnam-cat, MF319637-Vietnam-cat, MF319636-Vietnam-cat,
MF319633-Vietnam-cat
